# Supplementary figures and images for: A novel assay to isolate and quantify third-stage Dirofilaria immitis and Brugia malayi larvae emerging from individual Aedes aegypti
Source: Parasit Vectors. 2021 Jan 7;14:30. doi: 10.1186/s13071-020-04529-w (PMC7789620; doi:10.1186/s13071-020-04529-w)

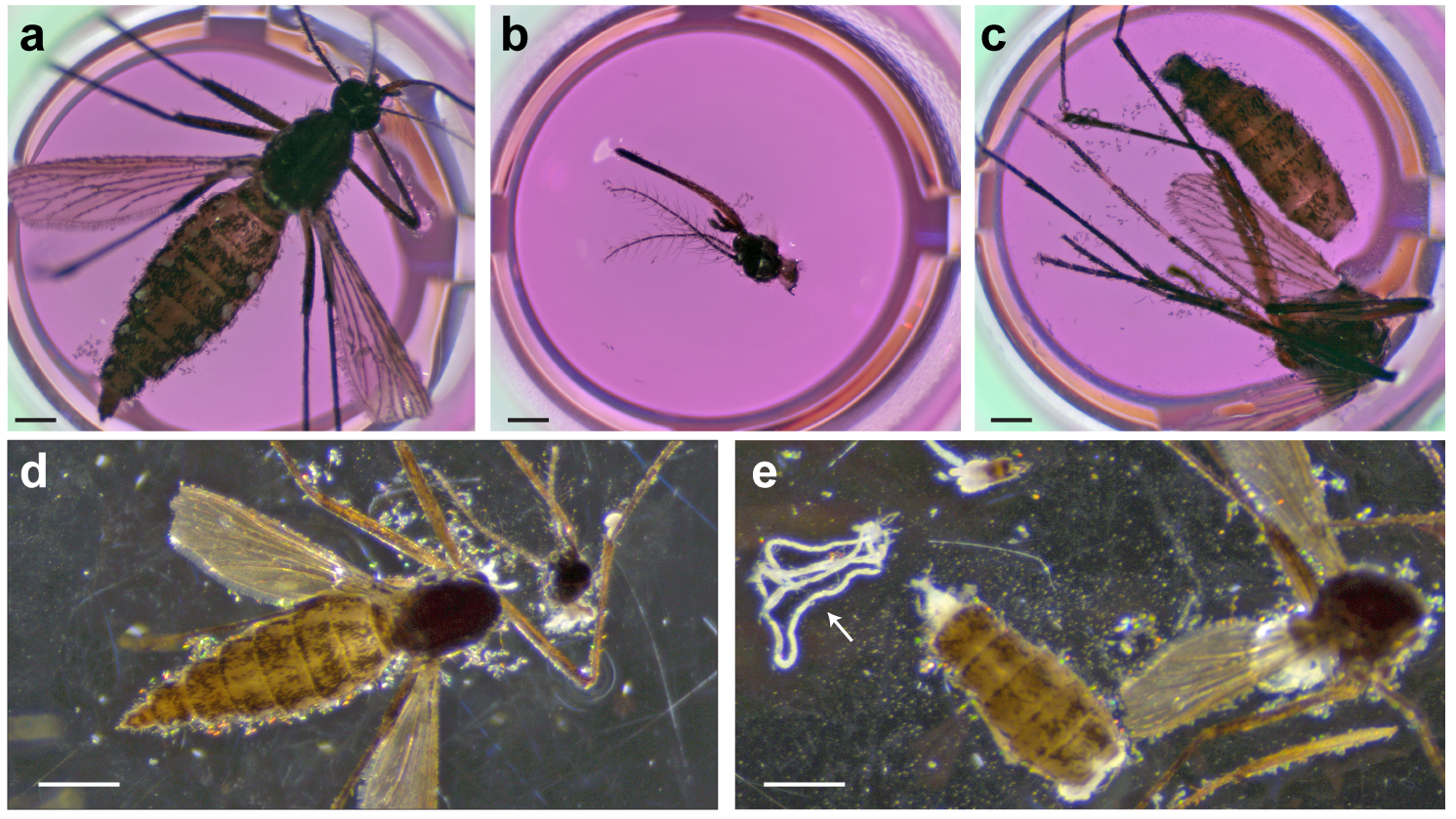

Supplement: Supplementary file 2 — Additional file 2: Figure S1. Mosquito dissection for analysis after emergence assay. After the emergence assay from the whole body (a), the head (b) and carcass (c) were placed individually into separate wells. The number of L3 larvae emerging from the dissected head and carcass were assayed after incubation at 37 °C. The Malpighian tubules were removed from the carcass prior to placing it in the well. Mosquito after removing head with fine forceps (d) and carcass after the Malpighian tubules (arrow) are dissected out (e). The scale bars in a–c and d, e are 500 and 1000 µm, respectively. [file 13071_2020_4529_MOESM2_ESM.tif]

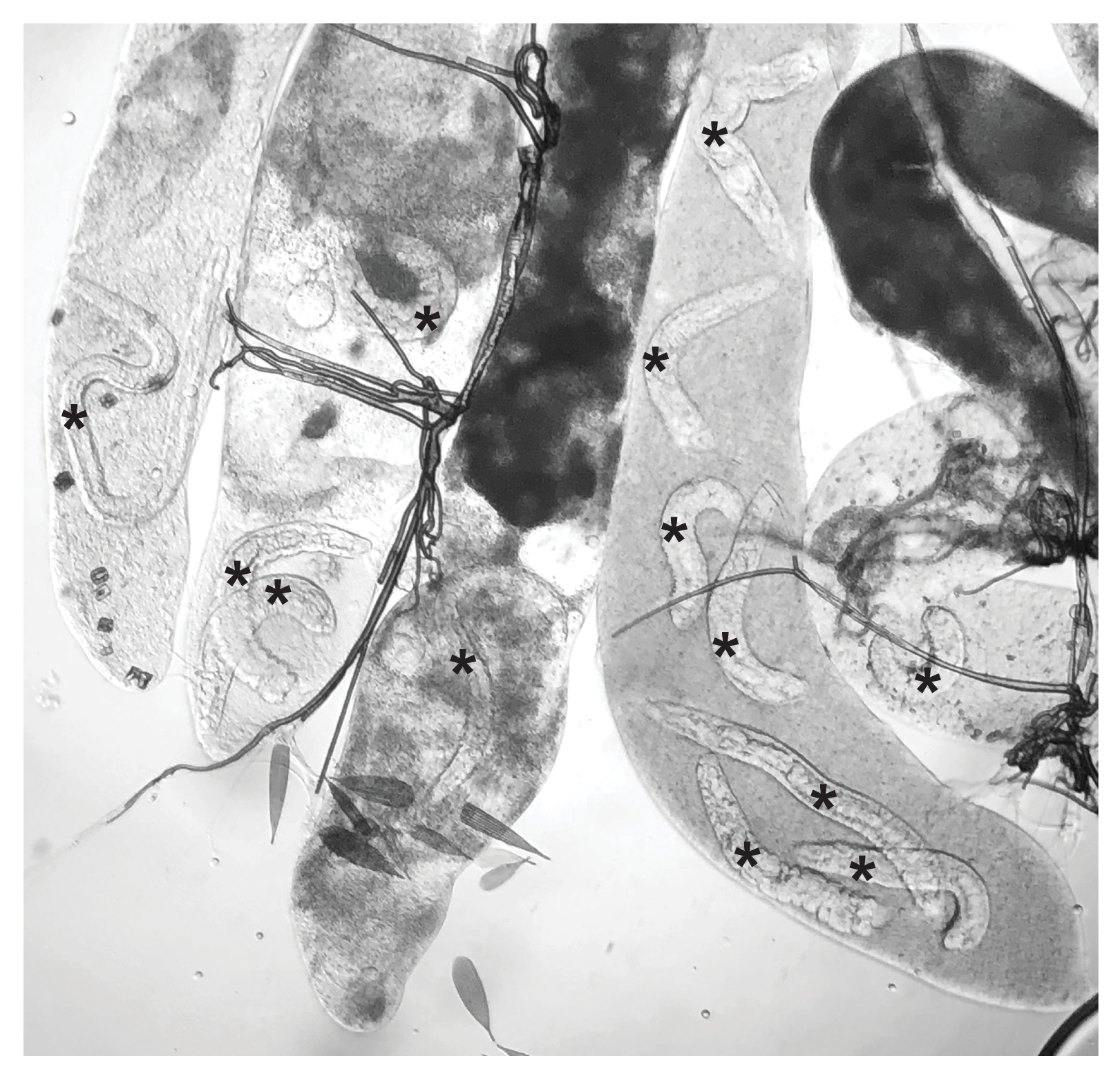

Supplement: Supplementary file 4 — Additional file 4: Figure S2. Example of D. immitis larval lengths scored in Malpighian tubules. Malpighian tubules dissected following an emergence assay contain viable larvae of different lengths indicated with asterisks. Different stage larvae are typically present. [file 13071_2020_4529_MOESM4_ESM.tif]

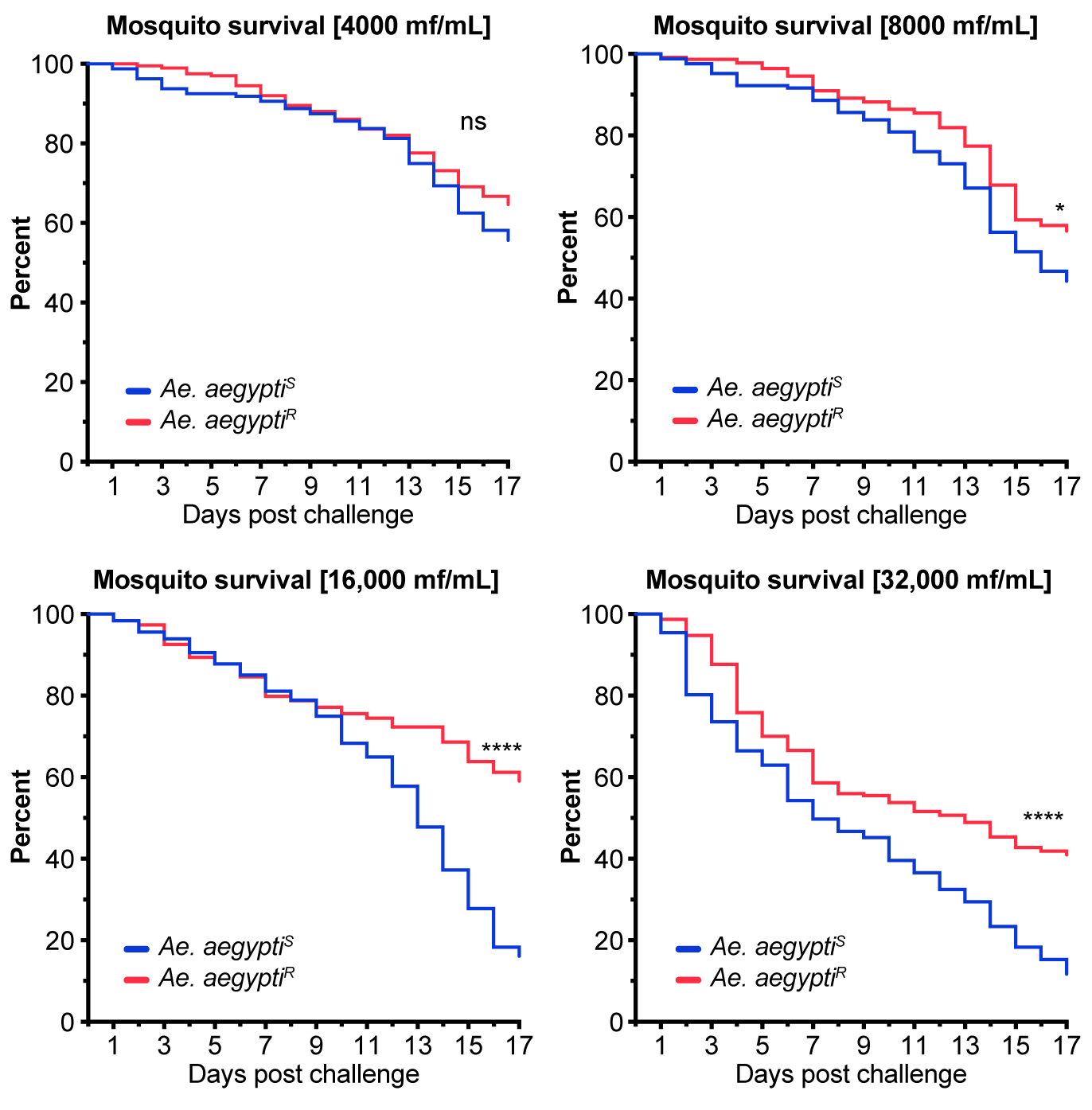

Supplement: Supplementary file 5 — Additional file 5: Figure S3. Ae. aegyptiS have greater mortality than Ae. aegyptiR following D. immitis infection. Pairs of adjacent treatment groups from Fig. 5d, e were analyzed by Kaplan–Meier and relationships, with significant differences indicated with asterisks here and in Additional file 6: Table S1. Aedes aegyptiS and Ae. aegyptiR are indicated in blue and red, respectively. [file 13071_2020_4529_MOESM5_ESM.tif]
