# Supplementary material for: A novel assay to isolate and quantify third-stage Dirofilaria immitis and Brugia malayi larvae emerging from individual Aedes aegypti
Source: Parasit Vectors. 2021 Jan 7;14:30. doi: 10.1186/s13071-020-04529-w (PMC7789620; doi:10.1186/s13071-020-04529-w)
Supplement: Supplementary file 6 — Additional file 6: Table S1. Pairwise comparisons of mosquito survival following blood feeding on different doses of D. immitis microfilariae. [file 13071_2020_4529_MOESM6_ESM.docx]

**Table S1.** Pairwise comparisons of mosquito survival following blood feeding on different doses of *D. immitis* microfilariae.

| ***Ae. aegypti^S^*** | **P-value** |
| --- | --- |
| 4k vs Blood feeding | <0.0001 |
| 8k vs 4k | 0.0337 |
| 16k vs 8k | <0.0001 |
| 32k vs 16k | <0.0001 |
| ***Ae. aegypti^R^*** | **P-value** |
| 4k vs Blood feeding | 0.0142 |
| 8k vs 4k | 0.1271 |
| 16k vs 8k | ns |
| 32k vs 16k | <0.0001 |
| ***Ae. aegypti^S^* vs *Ae. aegypti^R^*** | **P-value** |
| Blood feeding | \| ns (0.0798) \| \| --- \| |
| 4k | ns (0.1048) |
| 8k | \| 0.0113 \| \| --- \| |
| 16k | \| <0.0001 \| \| --- \| |
| 32k | \| <0.0001 \| \| --- \| |

Wilcoxon log rank survival test P-value shown for adjacent doses for *D. immitis* infection susceptible (*Ae. aegypti^S^*) and refractory (*Ae. aegypti^R^*) mosquitoes or between the same dose between the strains shown in Fig. 5d panels 5e.
